# 3′UTR Mapping Reveals Alternative Polyadenylation in Right Ventricular Failure

**DOI:** 10.1161/CIRCRESAHA.125.327629

**Published:** 2026-02-25

**Authors:** Rahul Neupane, Kartiga Natarajan, Erik E. Suarez, Henry J. Pownall, Kai-Lieh Huang, Eric J. Wagner, Ajay Kumar Verma, Rambabu Majji, Hari Krishna Yalamanchili, Ashrith Guha, Rajarajan A Thandavarayan

**Affiliations:** Department of Cardiology, DeBakey Heart and Vascular Center (R.N., K.N., E.E.S., A.G., R.A.T.), Houston Methodist Hospital, TX.; Department of Medicine (H.J.P.), Houston Methodist Hospital, TX.; Department of Biochemistry and Biophysics, University of Rochester Medical Center, NY (K.-L.H., E.J.W.).; Department of Pediatrics (A.K.V., R.M., H.K.Y.), Baylor College of Medicine, Houston, TX.; Department of Pediatrics, USDA/ARS Children’s Nutrition Research Center (H.K.Y.), Baylor College of Medicine, Houston, TX.; Jan and Dan Duncan Neurological Research Institute, Texas Children’s Hospital, Houston (H.K.Y.).

**Keywords:** 3′ untranslated regions, fibrosis, gene expression, polyadenylation, pulmonary hypertension, right ventricle

**Meet the First Author, see p e000751**

Right ventricular (RV) failure (RVF) is a major determinant of mortality in pulmonary hypertension,^1^ yet its molecular basis is unclear. Although RV adaptation to pressure overload involves distinct gene expression and structural remodeling, the posttranscriptional mechanisms driving this process remain poorly defined, including alternative polyadenylation (APA), a regulatory process that influences translation by selecting distinct polyadenylation sites (PAS) within a transcript.

## Human Tissue and Sequencing

RV tissue was obtained from 4 World Health Organization group I pulmonary hypertension patients (mean age 45, all female) undergoing heart–lung transplantation and 4 donor hearts. All had severe RV dysfunction and pulmonary artery systolic pressures of 70 to 140 mm Hg. Poly A-Click (PAC)-seq libraries were generated from snap-frozen RV tissue and analyzed using PolyA-miner.^2^ All human tissue collection and experimental procedures were performed in accordance with protocols approved by the Houston Methodist Hospital institutional review board. Findings were interpreted cautiously due to the limited cohort.

## APA Landscape of Failing Human RV

Global PAC-seq profiling revealed extensive 3′ untranslated region (3′UTR) remodeling, dominated by shortening (509 shortened versus 140 lengthened transcripts; Figure [A] and [B]). The poly(A) index summarizes APA shifts as the log_2_ ratio of proximal versus distal PAS usage; negative values indicate shortening, positive values lengthening. Shortened transcripts were enriched for transforming growth factor-β (TGF-β) signaling, myogenesis, and oxidative phosphorylation, including *TGFB1*, *tropomyosin 3*, *selenoprotein S* and *tribbles pseudokinase 2* genes linked to extracellular matrix (ECM) deposition and fibroblast activation. Few pathways were linked to lengthened 3′UTRs, underscoring the dominance of shortening in RVF. Comparison with left ventricular APA maps (Creemers et al^3^) showed minimal overlap (<4%), confirming RV-specific APA remodeling (Figure [C]).

**Figure.**
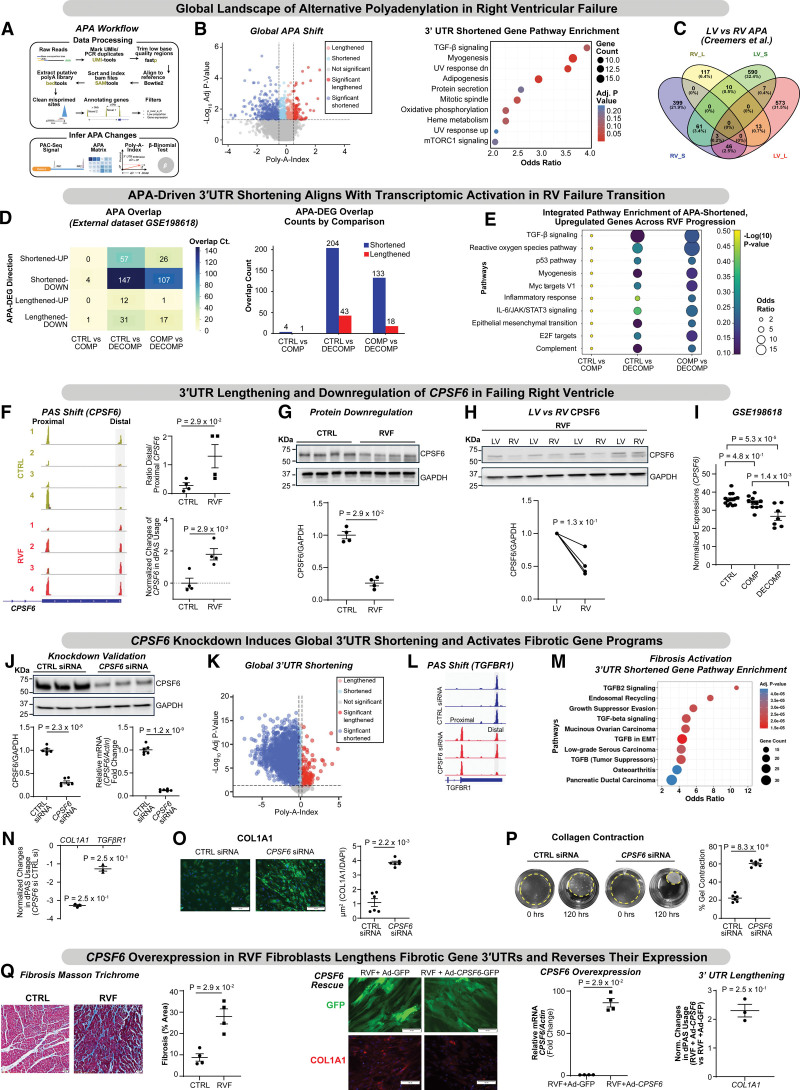
**Alternative polyadenylation (APA) remodeling drives right ventricular (RV) failure progression through *CPSF6* (cleavage and polyadenylation specific factor 6)-dependent 3′untranslated region (3′UTR) regulation: A and B.** Schematic of the alternative APA analysis workflow used to identify polyadenylation sites and quantify APA. Poly(A)-ClickSeq (PAC-seq) profiling of human RV tissue reveals widespread APA remodeling in end-stage RV failure, dominated by 3′UTR shortening (509 shortened, 140 lengthened transcripts). Pathway enrichment analysis of shortened transcripts is shown. **C**, Comparison with the published left ventricular (LV) APA data set (Creemers et al) demonstrates minimal overlap between RV-shortened, RV-lengthened, LV-shortened, and LV-lengthened gene sets (<4% shared), supporting chamber-specific RNA 3′-end remodeling. **D** and **E**, Integration with transcriptomic data (GSE198618) across control, compensated, and decompensated RV stages reveals APA activation only during decompensation—57 shortened-upregulated and 147 shortened-downregulated transcripts (control vs decompensated); similar trends in compensated vs decompensated. Enriched pathways include transforming growth factor β (TGF-β), epithelial–mesenchymal transition (EMT), inflammatory response, p53, and complement signaling. **F** through **I**, CPSF6, a core distal PAS regulator, shows 3′UTR lengthening and downregulation in failing RV by PAC-seq (genome browser view), quantitative polymerase chain reaction (qPCR; distal PAS usage), and Western blot. Paired LV–RV samples and external validation (GSE198618) confirm RV-specific CPSF6 suppression during decompensation. **J** through **P**, *CPSF6* knockdown in human cardiac fibroblasts induces 3′UTR shortening, activates TGF-β/ECM (extracellular matrix) pathways, increases COL1A1 (collagen type I alpha 1 chain) protein, and enhances contractility. **Q**, *CPSF6* overexpression in primary right ventricular failure (RVF) fibroblasts restores distal PAS usage, lengthens fibrotic gene 3′UTRs, and reduces COL1A1. Data are mean±SEM. Statistical tests: Beta-Binomial test (**B** and **K**), unpaired 2-tailed *t* test (**J** and **P**), Mann-Whitney *U* test (**F**, **G**, **O**, and **Q**), 1-sample Wilcoxon test vs 0 (**N** and **Q**), Wilcoxon matched‑pairs signed‑rank test (**H**), and 1-way ANOVA with Tukey test (**I**).

## APA Remodeling Aligns Specifically With the Transition to Decompensated RV Failure

To determine whether APA activation marks early adaptation or decompensation, we integrated PAC-seq data with the GSE198618 cohort (control, compensated RVH, decompensated RVF). No APA– differentially expressed gene (DEG) overlap occurred in control versus compensated hearts, but robust overlap emerged in decompensated RVs (57 shortened–upregulated; 147 shortened–downregulated genes; Figure [D] and [E]). Pathway analysis highlighted TGF-β signaling, interleukin ‑6/Janus kinase/signal transducer and activator of transcription 3, epithelial–mesenchymal transition, and inflammatory response. These findings indicate APA remodeling is a decompensation-specific program linked to fibrosis and inflammation.

## CPSF6 Is Selectively Downregulated and Associated With 3′UTR Lengthening in Failing Human RV

Poly(A) site mapping from RV PAC-seq data set revealed a consistent shift toward distal PAS usage in the *CPSF6* (cleavage and polyadenylation specificity factor 6) transcript, visualized in the University of California, Santa Cruz (UCSC) browser as a clear transition from proximal to distal polyA peaks (Figure [F]). This 3′UTR lengthening was validated by dPAS usage assays, confirming increased distal site utilization in failing RV. At the protein level, western blot analysis revealed marked CPSF6 reduction in failing RV compared with control hearts (Figure [G], bottom panel). Importantly, paired left ventricular and RV samples from the same patients demonstrated CPSF6 loss restricted to the RV, while left ventricular expression remained preserved (Figure [H], bottom panel). This paired-chamber comparison underscores *CPSF6* downregulation specificity to RVF.

Analysis of the external data set GSE198618 further supported these findings. *CPSF6* expression was unchanged between Control and Compensated RV but significantly decreased in both Control versus Decompensated and Compensated versus Decompensated comparisons (Figure [I]). This stepwise reduction across disease stages replicates our observations and identifies *CPSF6* downregulation as a robust molecular feature of RV decompensation. These data establish *CPSF6* suppression and 3′UTR lengthening as RV-specific events during transition to decompensated RVF.

## *CPSF6* Loss Is Sufficient to Induce Global 3′UTR Shortening and Activate Fibrosis Programs

To test causality, *CPSF6* was silenced in primary human cardiac fibroblasts. PAC-seq revealed widespread 3′UTR shortening affecting >2000 transcripts (Figure [J] and [K]). Profibrotic genes, including *TGFβR1* and *COL1A1* (collagen type I alpha 1 chain), showed marked shortening confirmed by PAC-seq and quantitative polymerase chain reaction, with UCSC browser analysis demonstrating a shift to proximal PAS. Pathway enrichment highlighted TGF-β signaling involvement. Shortened 3′UTRs correlated with higher COL1A1 and >60% collagen gel contraction versus ≈25% in controls (Figure [L] through [P]). *CPSF6* loss drives APA remodeling, promoting fibroblast transition and fibrosis via TGF-β activation.

## CPSF6 Overexpression Reverses Fibrotic 3′UTR Remodeling in Failing RV Fibroblasts

Primary fibroblasts from failing human RV tissue exhibited high collagen deposition and profibrotic marker expression (Figure [Q]). Adenoviral *CPSF6* delivery (Ad-CMV-*CPSF6*-GFP) elongated 3′UTRs of fibrotic transcripts and reduced COL1A1 protein compared with control (Ad-CMV-GFP), indicating that *CPSF6* restoration reverses pathogenic APA remodeling. Masson trichrome staining confirmed collagen-rich remodeling, underscoring *CPSF6*-dependent fibrosis regulation.

## Conclusions

APA remodeling is a defining feature of human RVF, involving both effector gene changes and disruption of core polyadenylation machinery. Despite a limited sample size, multi-level validation confirms biological relevance. Our RVF samples, obtained from rare end-stage pulmonary hypertension patients undergoing combined heart–lung transplantation, underscore the translational significance of these findings. Targeting APA or restoring *CPSF6* may be therapeutic, providing a framework for mechanistic and clinical studies.

## Article Information

### Data Availability

Poly(A)-clickseq (PAC-seq) data have been deposited in GEO under accession number GSE311052. Additional data and methods are available at https://doi.org/10.5281/zenodo.18272871.

### Disclosures

None.

## Supplementary Material


