# Supplementary material for: 3′UTR Mapping Reveals Alternative Polyadenylation in Right Ventricular Failure
Source: Circ Res. 2026 Feb 25;138(7):e327629. doi: 10.1161/CIRCRESAHA.125.327629 (PMC13015798; doi:10.1161/CIRCRESAHA.125.327629)
Supplement: Supplementary file 1 [file res-138-e327629-s001.pdf]

Major Resources Table

In order to allow validation and replication of experiments, all essential research materials listed in the Methods should be included in the Major Resources Table below. Authors are encouraged to use public repositories for protocols, data, code, and other materials and provide persistent identifiers and/or links to repositories when available. Authors may add or delete rows as needed.

Animals (in vivo studies)

Species	Vendor or Source	Background Strain	Sex	Persistent ID / URL

Genetically Modified Animals

	Species	Vendor or Source	Background Strain	Other Information	Persistent ID / URL
**Parent - Male**					
**Parent - Female**					

Antibodies

Target antigen	Vendor or Source	Catalog #	Working concentration	Persistent ID / URL
CPSF6	Proteintech	15489-1-AP	1:1000 (Western Blot)	https://www.ptglab.com/products/CPSF6-Antibody-15489-1-AP.htm?srsltid=AfmBOorqBhxN4wDVMyglysj28GEbvPHchW_B_16CbMsAJfTu-oRxn6qJ
GAPDH	Cell Signaling	2118L	1:1000 (Western Blot)	https://www.cellsignal.com/products/primary-antibodies/gapdh-14c10-rabbit-monoclonal-antibody/2118?srsltid=AfmBOor_o9rlIggfi-8oQEP2X6gugH0_FtDEceSFpvNe371-dkZ6bE9
Mouse anti-rabbit IgG	Santacruz	sc-2357	1:10000 (Western Blot)	https://www.scbt.com/p/mouse-anti-rabbit-igg-hrp?srsltid=AfmBOopRpyEmpsXosdjZBtWVHajhj_LeCfrKgBVYIq9OzBzh5IgJG2d
COL1A1	Cell Signaling	72026	1:200 (Immunofluorescence)	https://www.cellsignal.com/products/primary-antibodies/col1a1-e8f4l-rabbit-monoclonal-antibody/72026?srsltid=AfmBOoqNvZjQ

				ffmebFatpWzO5cRrdCT1LG4L1KT742P0-bhKEdJr24j7
Donkey anti rabbit IgG (H+L) Alexa Fluor 488	Thermo Fisher Scientific	A21206	2µg/ml (Immunofluorescence)	https://www.thermofisher.com/antibody/product/A21206.html?ef_id=EAIaIQobChMIq_-R9fmQkgMVuaADAB1ggA-9EAAYASAAEgI59fD_BwE:G:s&s_kwcid=AL!3652!3!!!!x!!!22626576456!&gad_source=1&gad_campaignid=22626577815&gbraid=0AAAAADxi_GTuIIHOdUw5HaL7Nn6j8e9ml&gclid=EAIaIQobChMIq_-R9fmQkgMVuaADAB1ggA-9EAAYASAAEgI59fD_BwE
Donkey anti-Rabbit IgG (H+L) Alexa Fluor™ 568	Thermo Fisher Scientific	A10042	4µg/ml (Immunofluorescence)	https://www.thermofisher.com/antibody/product/Donkey-anti-Rabbit-IgG-H-L-Highly-Cross-Adsorbed-Secondary-Antibody-Polyclonal/A10042

DNA/cDNA Clones

Clone Name	Sequence	Source / Repository	Persistent ID / URL

Cultured Cells

Name	Vendor or Source	Sex (F, M, or unknown)	Persistent ID / URL
Primary Human Cardiac Fibroblast	Promocell	unknown	https://promocell.com/us_en/human-cardiac-fibroblasts-hcf.html?srsltid=AfmBOor4w8BMkg-E5va-Or-RXhnEUqKhh9oEwgGWZbPbMKInC1YPGmKL
Patient derived cardiac fibroblasts from right	Explanted right ventricular tissue from patients with advanced	F	N/A

ventricular failure tissue	pulmonary hypertension undergoing combined heart–lung transplantation.		

Data & Code Availability

Description	Source / Repository	Persistent ID / URL
PAC seq data	GEO database	GSE311052 https://www.ncbi.nlm.nih.gov/geo/query/acc.cgi?acc=GSE311052
Other Underlying data	Zenodo	18272871 https://doi.org/10.5281/zenodo.18272871

Other

Description	Source / Repository	Persistent ID / URL
RNeasy Mini kit	Qiagen	74106
Qiazol	Qiagen	79306
qScript cDNA Synthesis Kit	Quantabio	95047-025
SsoAdvanced Universal SYBR Green Supermix	BioRad	1725274
T-PER Tissue Protein Extraction reagent	Thermo Fisher Scientific	78510
Halt Protease & Phosphatase Inhibitor Use Cocktail 100x	Thermo Fisher Scientific	1861280

RIPA buffer	Thermo Fisher Scientific	89900
BCA protein Assay	Thermo Fisher Scientific	23227
Clarity Western ECL Substrate	BioRad	1705061
4X Laemmli Sample buffer	Biorad	1610747
Mini Protean TGX Stain free gels	BioRad	4568083
10x Tris Glycine SDS buffer	BioRad	1610732
20x TBS buffer	Thermo Fisher Scientific	28358
Transblot Turbo 5x Transfer buffer	BiorRd	10026938
Precision Plus Protein Dual Color Standards	BioRad	1610374
Everyblot blocking buffer	BioRad	12010020
Transblot Turbo 5x Transfer buffer	BioRad	10026938
Restore plus western blot stripping buffer	Thermo Fisher Scientific	46430
Fibroblast Growth Medium 3	Promocell	C-23025
Pen Strep	Thermo Fisher Scientific	15070-063

TrypLE™ Express Enzyme (1X)	Thermo Fisher Scientific	12605010
Control adenovirus	Vector builder	pAV[Exp]-CMV>EGFP; Vector ID: VB010000-9299hac
CPSF6 adenovirus	Vector builder	pAV[Exp]-EGFPCMV>hCPSF6[NM_001300947.1]; Vector ID: VB900010-7552jxu
Human CPSF6 Forward Primer	Sigma Aldrich	AAGCTGAATATGGTGGGCATGA
Human CPSF6 Reverse Primer	Sigma Aldrich	TAATCTCGGTCTTCTGGGGC
Human β-actin Forward Primer	Sigma Aldrich	ATTGCCGACAGGATGCAGAA
Human β-actin Reverse Primer	Sigma Aldrich	GGGCCGGACTCGTCATACTC
Human CPSF6 long Forward Primer	Sigma Aldrich	ATGTCTAGGCTTCTGGGAGGA
Human CPSF6 long Reverse Primer	Sigma Aldrich	TCGCTAAATGCAGGGTCTGT
Human _COL1A1 Forward Primer	Sigma Aldrich	GTGCGATGACGTGATCTGTGA
Human _COL1A1 Reverse Primer	Sigma Aldrich	CGGTGGTTTCTTGGTCGGT
Human_COL1A1 long Forward Primer	Sigma Aldrich	GTGAGGGAGACAGACACCTG
Human_COL1A1 long Reverse Primer	Sigma Aldrich	GTGTTCTGGGGATTCAGGAG
Human_TGFβR1 Forward	Sigma Aldrich	ACGGCGTTACAGTGTTTCTG
Human_TGFβR1 reverse	Sigma Aldrich	GCACATACAAACGGCCTATCTC
Human_TGFβR1 long Forward	Sigma Aldrich	TTTGTGCAGGATTCTTTAGGCTT
Human_TGFβR1 long Reverse	Sigma Aldrich	GGCTTCTCAGTATCATTCGACTT
VECTASHIELD Vibrance® Antifade Mounting Medium with DAPI	Vector laboratories	H-1800-2
CPSF6 siRNA	Santacruz Biotechnology	sc-72990
Control siRNA	Santacruz Biotechnology	sc-44236

Lipofectamine RNAiMAX Transfection Reagent	Thermo Fisher Scientific	13778075
Optimem Media	Gibco	31985-062
Trichrome Stain (Masson) Kit	Sigma	HT15-1KT
Collagenase Type 4	Worthington biochemical corporation	LS004188
Dispase II	Sigma	D4693
DNase I	Sigma	D45131VL
DPBS with calcium/ magnesium	Thermo Fisher Scientific	14040133
DPBS with no calcium/ magnesium	Thermo Fisher Scientific	14190144
