# Supplementary material for: 3′UTR Mapping Reveals Alternative Polyadenylation in Right Ventricular Failure
Source: Circ Res. 2026 Feb 25;138(7):e327629. doi: 10.1161/CIRCRESAHA.125.327629 (PMC13015798; doi:10.1161/CIRCRESAHA.125.327629)
Supplement: Supplementary file 2 [file res-138-e327629-s002.pdf]

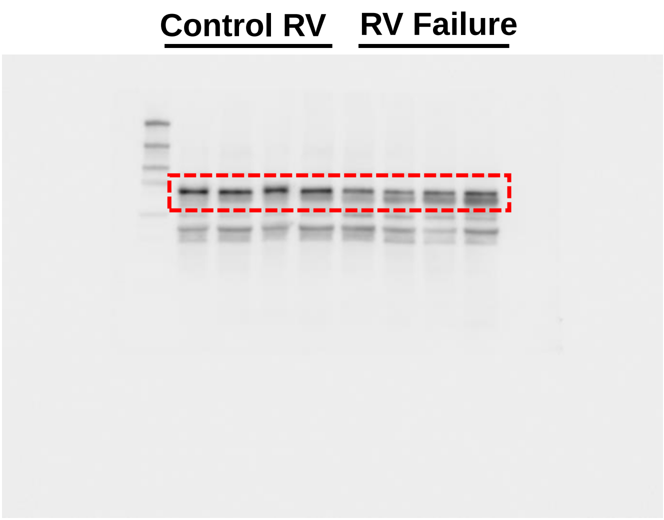

CPSF6 Full unedited gel

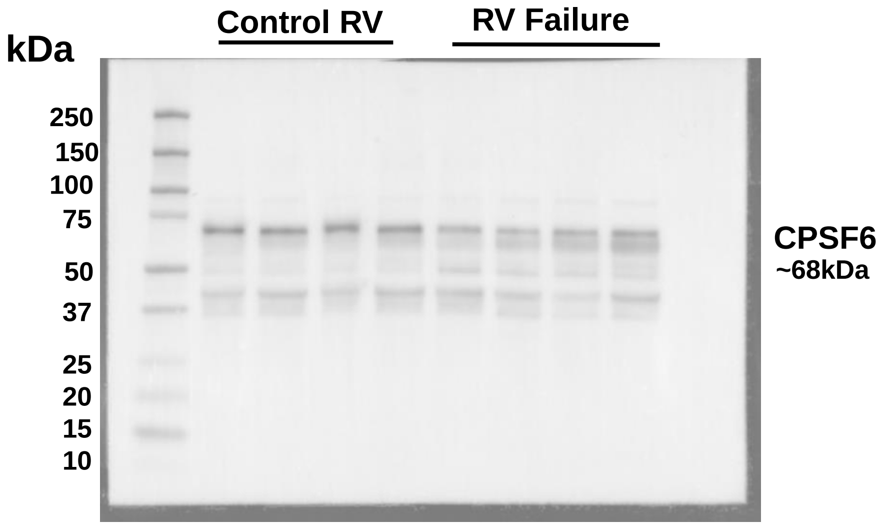

Colorimetric marker image overlaid on chemiluminescent blot from the same membrane

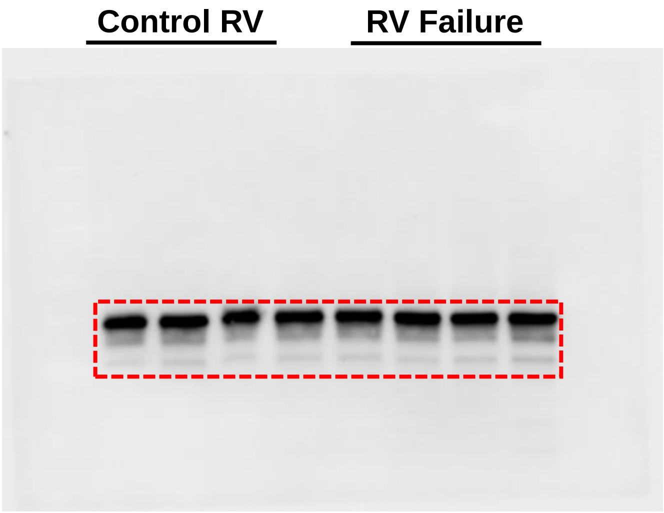

GAPDH Full unedited gel

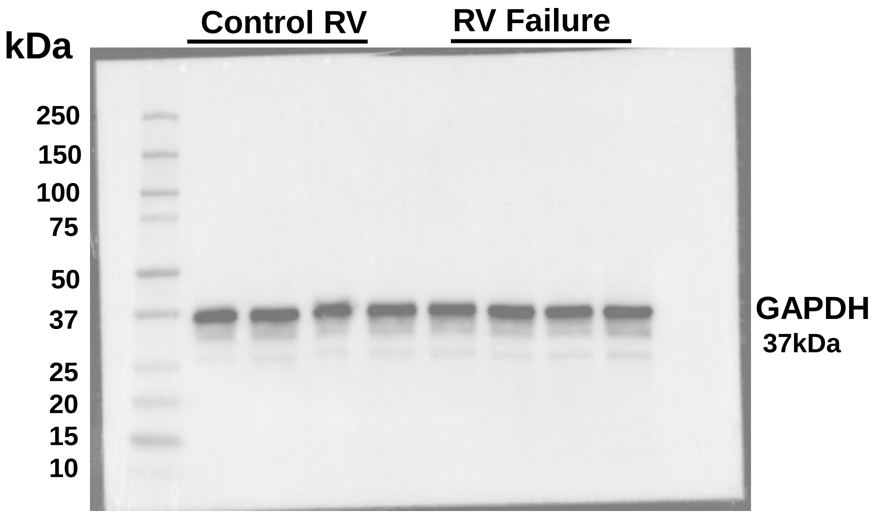

Colorimetric marker image overlaid on chemiluminescent blot from the same membrane

Full unedited blots of CPSF6 and GAPDH from Figure G. CPSF6 was stripped and probed on the same membrane of GAPDH following stripping and blocking. The red-dotted box indicates the blot region shown in the manuscript.

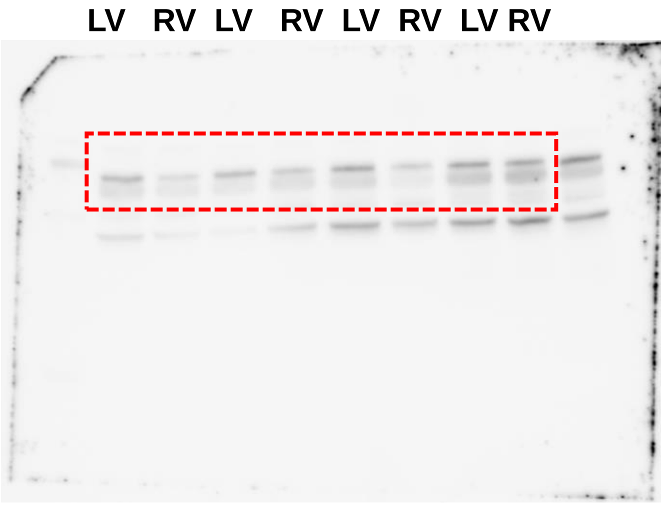

CPSF6 Full unedited gel   last lane is unrelated

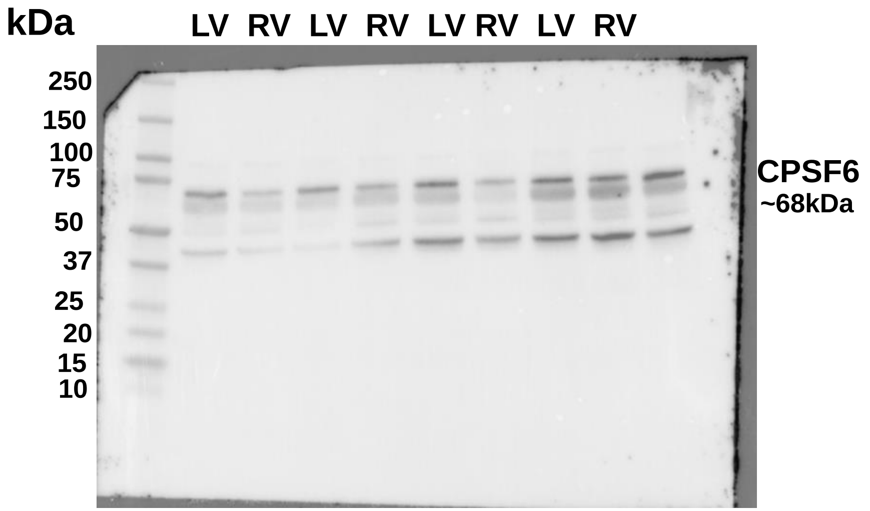

Colorimetric marker image overlaid on chemiluminescent blot from the same membrane

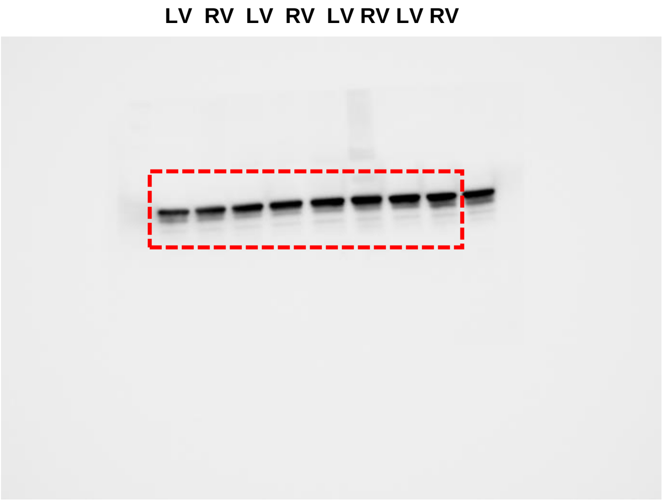

GAPDH Full unedited gel

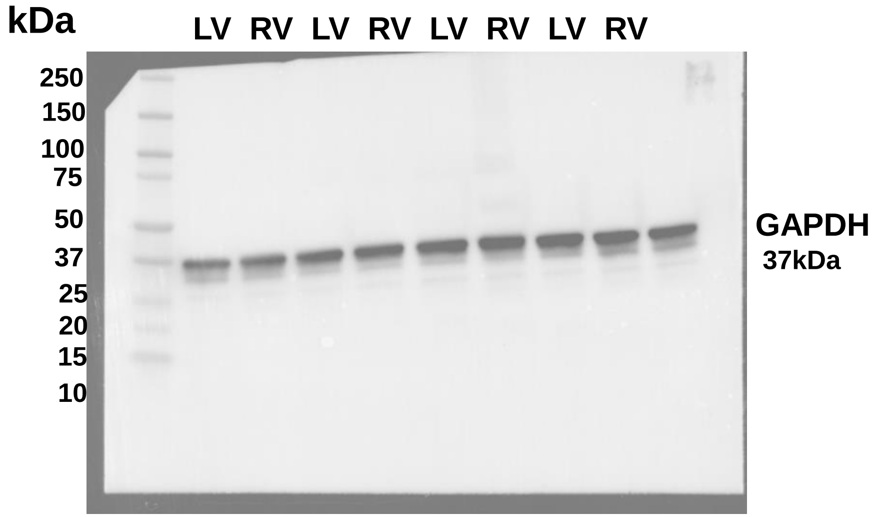

Colorimetric marker image overlaid on chemiluminescent blot from the same membrane

Full unedited blots of CPSF6 and GAPDH from Figure H . GAPDH was stripped and probed on the same membrane of CPSF6 following stripping and blocking. The red-dotted box indicates the blot region shown in the manuscript.

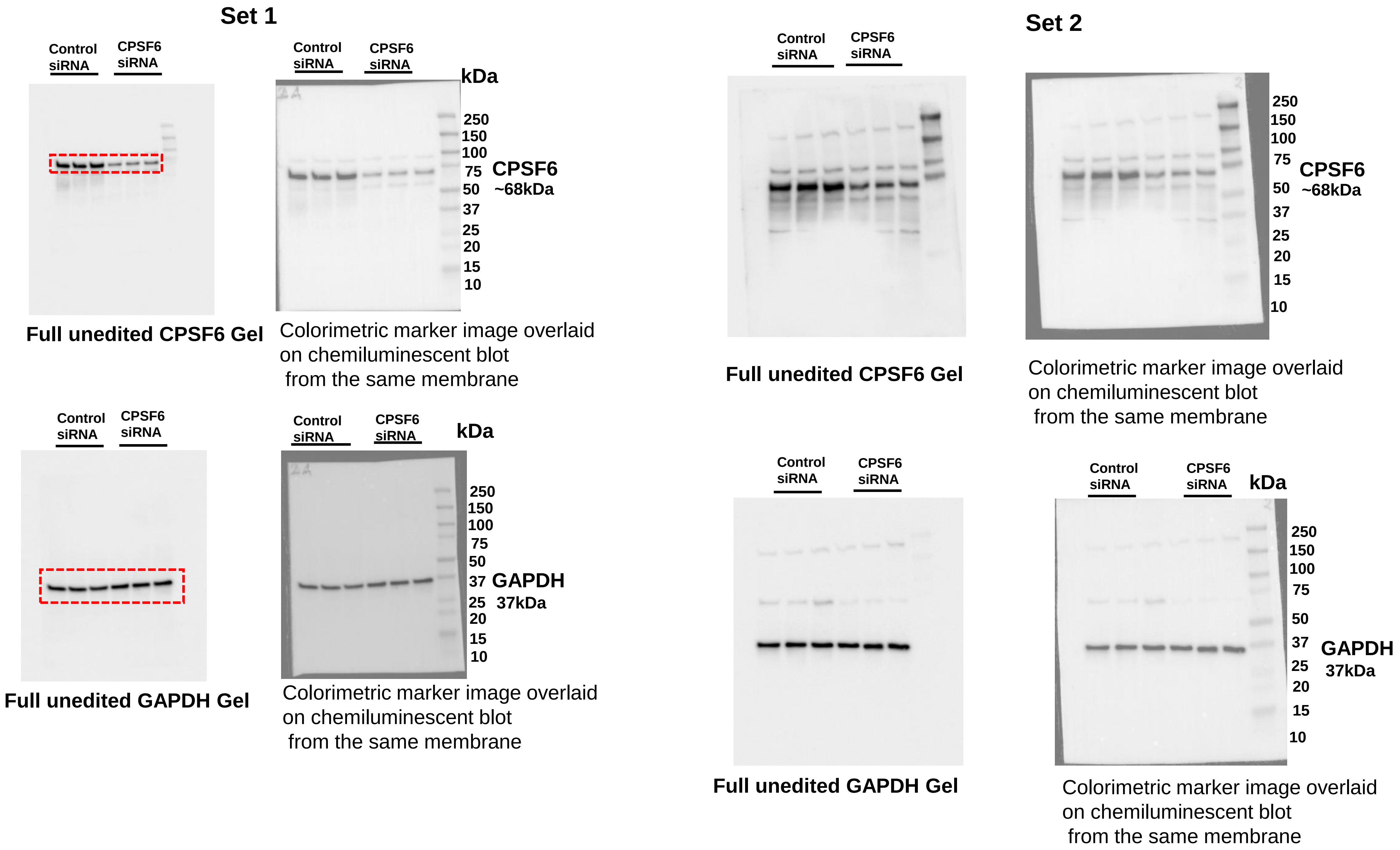

Full unedited blots of CPSF6 and GAPDH from Figure J . GAPDH/CPSF6 was stripped and probed on the same membrane following stripping and blocking. The red-dotted box indicates the blot region shown in the manuscript.
